# A case report of a novel compound heterozygous mutation in a Brazilian patient with deficiency of Interleukin-1 receptor antagonist (DIRA)

**DOI:** 10.1186/s12969-020-00454-5

**Published:** 2020-08-20

**Authors:** Leonardo Oliveira Mendonça, Alice Grossi, Francesco Caroli, Robson Aguiar de Oliveira, Jorge Kalil, Fabio Fernandes Morato Castro, Alessandra Pontillo, Isabella Ceccherini, Myrthes Anna Maragna Toledo Barros, Marco Gattorno

**Affiliations:** 1 Autoimmune and Autoinflammatory Unit; Clinical Immunology and Allergy Department, School of Medicine, University of São Paulo, Rua Dr. Enéas de Carvalho Aguiar, 255 – 8 Andar, Sao Paulo, Brazil; 2 UOSD Genetics and Genomics of Rare Diseases, Istituto Giannina Gaslini, Genoa, Italy; 3 Immunogenetic Laboratory, Department of Immunology, Biomedical Science Insitute, University of Sao Paulo, Sao Paulo, Brazil; 4 Center for Autoinflammatory Diseases and Immunodeficiencies, IRCCS Giannina Gaslini, Genoa, Italy

**Keywords:** DIRA, Deficiency of interleukin-1 receptor antagonist, IL1RN

## Abstract

**Background:**

Deficiency of the natural antagonist of interleukin-1 was first described in 2009 and so far 20 patients has been reported. In Brazil just two cases have been reported both carrying the same homozygous 15 bp deletion. Blocking interleukin-1 has changed rate survival for DIRA patients. The use of anakinra and rilonacept has been reported safe and efficient, whereas the selective blockade of interleukin-1 beta, using the monoclonal antibody canakinumab has been reported in a single case only.

**Case presentation:**

Here we report a case of a 7 years old Brazilian boy that presented with recurrent episodes of systemic inflammation with severe disabling osteomyelitis with mild pustular skin rash. A Next Generation Sequencing gene panel allowed to detect two pathogenic mutations in the IL1RN gene, described in compound heterozygosity. Corticosteroids was effective in controlling inflammation and anti-IL1 beta blocker triggered disease flare. Complete clinical control could be achieved using IL-1 receptor antagonist.

**Conclusions:**

DIRA is a severe, life threatening autoinflammatory condition with low numbers of patients described all over the world. The mutation p.Asp72_Ile76del in IL1RN is presented in all Brazilian DIRA patients already described and p.Q45* (rs1019766125) is a new mutation affecting the IL1RN gene. Following the pathogenesis of DIRA, blocking both subunits of interleukin one as well as antagonizing the receptor using anakinra or rilonacept seems to be effective. There is just one report using canakinumab for the treatment of DIRA and this is the first report of disease flare using this drug.

## Background

Autoinflammatory diseases are a group of rare disorders typically characterized by fever and systemic organ-specific inflammation. In 1999, Daniel Kastner introduced the current concept that recognized innate immune system as responsible for the immune dysregulation at that time in two conditions, Familial Mediterranean fever (FMF) and TNF receptor associated periodic syndrome (TRAPS) [1].

Deficiency of interleukin (IL)-1 receptor antagonist (DIRA; MIM: 612852) was firstly described in 2009 by Aksentijevich et al., as a rare autoinflammatory disease with a very early onset, systemic inflammation (high level of acute-phase reactants) and marked skin and bone involvement [2]. DIRA is an autosomal recessive syndrome due to loss-of-function mutations in the IL-1 receptor antagonist/IL1RA gene (*ILR1N*). IL1RA is secreted by a variety of immune and not-immune cells and binds non-productively to cell surface IL-1 receptor (IL1R), preventing IL-1ß and IL-1α pro-inflammatory effect. It contributes to the control of inflammatory process, and its loss of function results in continuous pro-inflammatory signaling [3].

Since 2009, at least 18 distinct mutations localized along the entire *IL1RN* gene have been described (https://fmf.igh.cnrs.fr/ISSAID/infevers/) in patients from different countries. The involvement of IL-1 in the pathogenesis of DIRA prompted to the use of IL-1 inhibitors (i.e.: anakinra, rilonacept and canakinumab) with dramatic results and complete remission of the symptoms as previously reported [2, 3].

This study reports a novel case of a Brazilian DIRA patient with two biallelic mutations, and its clinical response to different anti-IL1 drugs.

## Case report

**Fig. 1:**
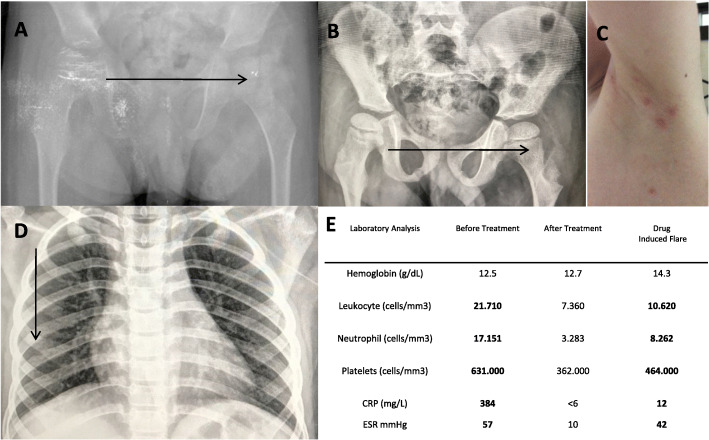
Clinical follow up. Femur x-ray before (**a**) and after treatment (**b**) with narrows indicating resolution of severe osteomyelitic lesions and (**c**) with an initial pustular rash in the forearms observed. The thoracic X-Ray (**d**) with black narrow indicating an acute osteomyelitis in the 7th rib during a flare induced by canakinumab. The figure (**e**) shows laboratory control of acute reactant markers before and after the treatment and the inflammation induced by the drug induced flare

A 7-year-old Brazilian child came to our attention because of recurrent severe osteomyelitis episodes since the 4th month of life, associated with systemic inflammation and/or skin rash (Fig. 1). Clinical and laboratory investigations were performed ruling out both infections and primary immunodeficiency and the clinical suspicion of a DIRA was pointed out. Treatment with 1 mg/kg prednisolone was started with resolution of the pustular rash and significantly reduction on inflammatory markers. However, persistent pain on left hip and radiological osteomyelitis was observed.

**Fig. 2:**
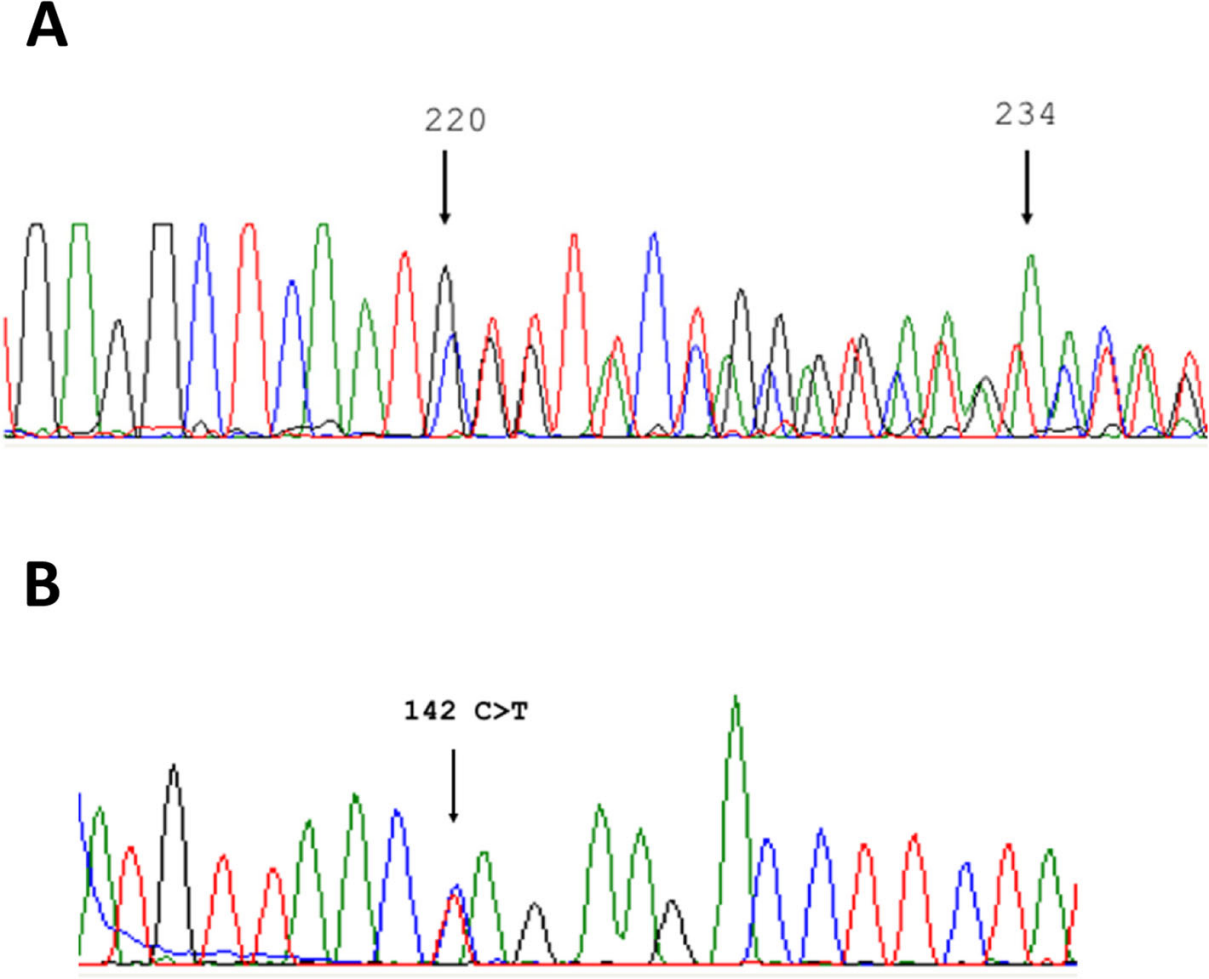
DNA sequence electropherograms demonstrating the p.Ile71_Pro75del and p.Gln45Ter heterozygous mutations in *IL1RN*. Sanger sequencing results for *IL1RN* mutations in DIRA patient. Electropherograms of the two mutations are reported. **a** p.Ile71_Pro75del, reverse strand and (**b**) p.Gln45Ter, forward strand. Black arrows indicated (A) the first and last nucleotides of the deletion; **b** the nucleotide mutated

After 6 months of follow up, an anti-IL-1 therapy was started. Due to the major facility to obtain canakinumab in Brasil, the anti-IL1ß monoclonal antibody was started at the dose of 2 mg/kg/month, with a initial control of the pain on left hip and corticosteroids could be tapered to 0,5 mg/kg. However, immediately after the seventh dose the patient presented thoracic pain with elevation of the acute reactants markers. A new osteomyelitis in the fifth rib was detected (Fig. 1) and corticosteroid dose was returned to 1 mg/kg. Therefore, at the age of 8 years, the treatment was switched to anti-IL1 receptor antagonist (IL-1Ra, anakinra) at the dose of 2 mg/kg/day. After 1 month on anakinra, marked control of skin rash, bone pain, inflammatory markers and radiological osteomyelitis was a observed (Fig. 2). After 18 months of treatment with anakinra, corticosteroids could be discontinued and the patient is completely free of bone and skin manifestations and his laboratory examinations are persistently in the normal range.

### Genetic analysis

Genomic DNA of the patient and his parents was sent to the Laboratory of Medical Genetics of the “Giannina Gaslini” Institute (Genova, Italy) for the analysis of the next generation sequencing (NGS) based autoinflammatory gene panel described in Rusmini et al., ARD 2015, including 10 genes: IL1RN, LPIN2, MEFV, MVK, NLRP12, NLRP3, NOD2, PSTPIP1, PSMB8, and TNFRSF1A. Mutation search was carried out using a library of amplicons suitable to capture the coding portion, and short flanking intronic sequences, of the above 10 genes, as designed through the Ampliseq online tool [4], coupled with the Ion PGM™ parallel sequencing (Thermofisher Scientific). Such a genetic screening demonstrated the presence of two already described mutations in the *IL1RN gene* found, for the first time, in compound heterozygosity*:* p.Ile71_Pro75del (NM_173842.2: c.211_225del leading to p.I71_P75del) and p.Gln45Ter (NM_173842.2: c.133C > T leading to p.Q45*, rs1019766125). These mutations were confirmed by Sanger sequencing as reported in Figure 3 (primer sequences are reported in Supplementary File). The deletion, p.I71_P75del was found in the mother whereas the p.Q45* is a de novo mutation.

## Discussion

Bone autoinflammatory disorders are a group of inflammatory diseases caused by the unprovoked activation of the innate immune system leading to bone inflammatory process mediated mainly by constitutive overproduction of interleukin 1-β. In these disorders the inflammatory lesions are osteolytic lesions characterized by persistent chronic inflammation with recurrent periods, and a typical negative microbiologic culture, and a non-specific histopathologic feature [3, 5].

The so far described autoinflammatory bone disorders in childhood include: 1 - chronic recurrent multifocal osteomyelitis (CRMO; OMIM: 259680); 2 – deficiency of interleukin-1 receptor antagonist (DIRA; OMIM: 612852), also known as Osteomyelitis, Sterile Multifocal, with Periostitis And Pustulosis (OMPP); 3 - synovitis–acne–pustulosis–hyperostosis–osteitis (SAPHO Syndrome); 4 - Chronic Recurrent Multifocal Osteomyelitis, Congenital Dyserythropoietic Anemia, And Neutrophilic Dermatosis (Majeed’s syndrome, MJDS; OMIM: 609628); 5 - arthritis-related syndromes associated with pyoderma gangrenosum (PAPA, OMIM: 604416) [5]. DIRA is potentially fatal, and may be confused with neonatal sepsis. If not diagnosed or properly treated, the estimated rate of death in DIRA is about 7 in 24 patientes. (Table 1).

**Table 1:** Summary of DIRA patients already diagnosed, world demographic distribution, mutations and outcomes. On B, specific mutations found in Brazilian DIRA patients

Mutation	Country	Number of patients	Death	Treatment	Reference
**A**					
c.-4153_191del; del IL1RN	India	1	0	Anakinra	8
c.62C > G; p.Ser21*	Germany	1	0	Anakinra	9
c.76 C > T; p.(Arg26*)	Turkey	1	0	Canakinumab	10
c.156_157del; p.(Asn52Lysfs*25)	Canada	1	0	Anakinra	8
c.160C > T; p.(Gln54*)	Lebanon	2	0	Anakinra	8
c.229G > T; p.(Glu77*)	Netherlands	5	3	Anakinra	8
c.355C > T; p (Gln119*)	Turkey	2	2	Not treated	8
c.396delC; p(Thr133Profs*118)	Turkey	1	0	Anakinra	8
IL1RN locus deletion	Puerto Rico	4	1	Anakinra	8
c.229G > T; p.Gly77* and c.140delC	USA	1	0	Anakinra	8
**B**					
c.213_227del; p.Asp72_Ile76del	Brazil	2	0	Anakinra	8
c.211_225del; p.I71_P75del and c.133C > T; p.Q45*	Brazil	1	0	Anakinra	This patient

In DIRA, the clinical presentation feature includes generalized pustulosis (marked in the upper forehead and back), bone inflammation (usually osteitis and periostitis) and systemic inflammation. Within the first few weeks after birth, a pustular eruption and systemic signs of inflammation develops. Although systemic inflammatory markers are markedly elevated, fever is usually absent. Weeks after the rash occurs, osteitis is detected. The osteitis seen in DIRA is severe, with ample bone involvement, well-marked osteolytic and periosteum patterns. These bone lesions typically affect the long bones, vertebral bodies and have a predilection for the proximal femur. Bone biopsy in DIRA is characterized by a sterile purulent osteomyelitis (negative microbiologic culture), fibrosis and sclerosis [2].

In Brazil, the first recognized patients with DIRA were two unrelated Brazilian children carrying the same novel homozygous 15-bp (in-frame) deletion on the *IL1RN* gene (p.Asp72_Ile76del) [3]. Previously, only one DIRA patient with a compound heterozygosity in *IL1RN* gene has been reported in the USA (p.E77* and c.140delC leading to p.T47TfsX4) [6]. Our patient is the second patient carrying a compound heterozygous mutation affecting the IL1RN gene. Curiously, our patient carries in one allele the same deletion already described in Brazilian DIRA patients whereas in the other allele a new mutation affecting the IL1RN gene was found. Consequently, both can be founder mutations for DIRA in Brazil (Table 1).

The development of anti-IL1 drugs (anakinra, canakinumab and rilonacept) improved rate survival in DIRA [2]. Recently, Rilonacept has also demonstrated inflammation remission in DIRA patients [7]. There is just one published report of positive use of canakinumab in DIRA. However the follow-up was of some months only, with no detail on the long-term outcome [8]. Of note, since canakinumab blocks specifically the serum free IL1β, it is possible that the accumulation of IL1α may lead to independently activate the natural receptor. Indeed, the introduction of recombinant IL-1Ra allowed a complete control of disease activity, as reported on Table 1  [9–11].

## Conclusion

DIRA is a rare genetic condition with early onset and severe prognosis if not properly recognized and treated. The low number of confirmed cases may reveal DIRA’s misdiagnosis. We report the first compound heterozygous mutation in IL1RN gene in a Brazilian’s DIRA patient. This is the first report of p.Q45* mutation affecting the IL1RN gene. All the Brazilian patients already described seems to be affected by the deletion here described, p.Asp72_Ile76del.

## Supplementary information

**Additional file 1.** Genetic segregation in the mother and the father of mutations found. Forward and Reverse primmers used in exons 2 and 3 of the IL1RN gene.

## Data Availability

The datasets used and/or analysed during the current study are available from the corresponding author on reasonable request.
