# Supplementary material for: A case report of a novel compound heterozygous mutation in a Brazilian patient with deficiency of Interleukin-1 receptor antagonist (DIRA)
Source: Pediatr Rheumatol Online J. 2020 Aug 20;18:67. doi: 10.1186/s12969-020-00454-5 (PMC7439677; doi:10.1186/s12969-020-00454-5)
Supplement: Supplementary file 1 — Additional file 1. Genetic segregation in the mother and the father of mutations found. Forward and Reverse primmers used in exons 2 and 3 of the IL1RN gene. [file 12969_2020_454_MOESM1_ESM.pdf]

Appendix 1 – Genetic segregation in the mother and the father of mutations found

IL1RN; Ex 4; Father:
Wild Type

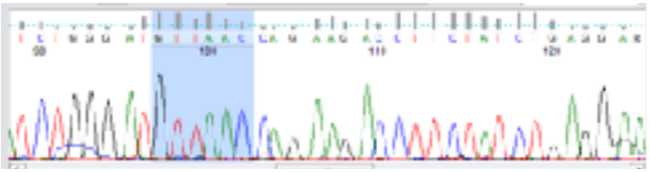

IL1RN; Ex 5; Father:
Wild Type

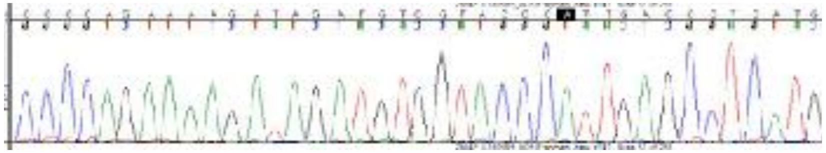

IL1RN; Ex 4; Mother:
Wild Type

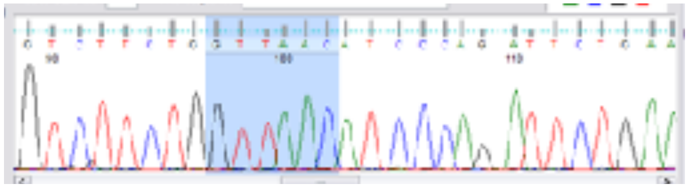

IL1RN; Ex 5; Mother:
c.220_234del15bp (p.Ile74_Pro78del)

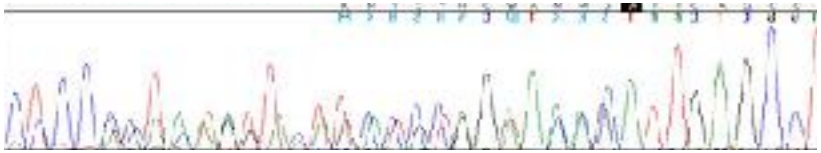

IL1RN; Ex 4;Patient:
De novo, c.142 C>T (p.Gln48Ter)

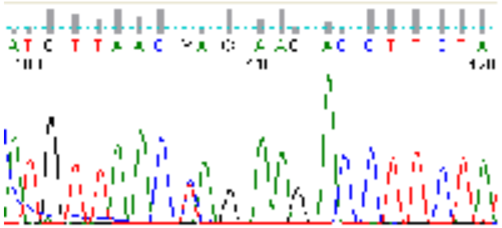

IL1RN Ex 5; Patient:
c.220_234del15bp (p.Ile74_Pro78del)

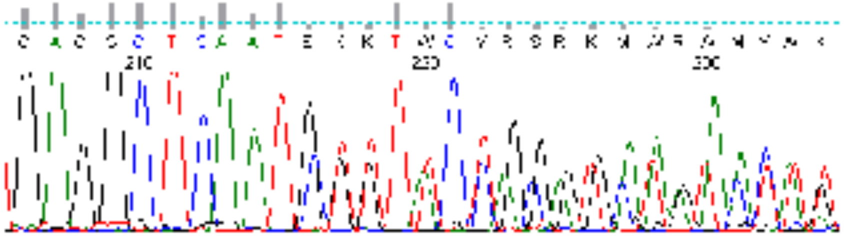

Appendix 1 – Forward and Reverse primmers used in exons 2 and 3 of the IL1RN gene

GENE	PRIMER F 5’->3’	PRIMER R 5’->3’
IL1RN EXON 2 (NM_173842.2)	CCTGGAAGAGCTGGATGCAA	AAGTGACGTGATGCCCACAT
IL1RN EXON 3 (NM_173842.2)	TAACCTGACCCTCCCCTCTG	AGCTTCCAAAGTGACCCCAG
